# Comparative omics reveals conserved extracellular matrix signatures during mammary tumor progression

**DOI:** 10.3389/fonc.2026.1788746

**Published:** 2026-03-24

**Authors:** Bruno Sousa de Almeida, Gisele Vieira Rocha, Simone Nunes, Dalila Luciola Zanette, Michel Batista, Alessandra Estrela-Lima, Carlos Gustavo Regis-Silva, Karine Araújo Damasceno

**Affiliations:** 1 Laboratory for Global Health Research and Neglected Diseases, Gonçalo Moniz Institute, Oswaldo Cruz Foundation, Fiocruz, Salvador, Bahia, Brazil; 2 D’Or Institute for Research and Education (IDOR), Salvador, Brazil; 3 Laboratory for Applied Science and Technology in Health, Carlos Chagas Institute, Oswaldo Cruz Foundation, Fiocruz, Curitiba, Paraná, Brazil; 4 Mass Spectrometry Platform, Carlos Chagas Institute (ICC), Fiocruz, Curitiba, Paraná, Brazil; 5 Research Center on Mammary Oncology/Hospital of Veterinary Medicine Professor Renato Medeiros Neto (NPqOM/HOSPMEV), Federal University of Bahia, Salvador, Bahia, Brazil; 6 Advanced Public Health Laboratory, Gonçalo Moniz Institute, Oswaldo Cruz Foundation, Fiocruz, Salvador, Bahia, Brazil

**Keywords:** Comparative oncology, extracellular matrix, mammary tumor, proteomics, tumor progression

## Abstract

The extracellular matrix (ECM) plays a critical role in tumor progression by modulating cell adhesion, migration, and signaling; however, its contribution to metastatic progression in spontaneous mammary tumors remains poorly understood. Mammary tumors are among the most common neoplasms in female dogs and share histopathological and molecular similarities with human breast cancer, supporting their use as a comparative oncology model. To investigate ECM remodeling during tumor progression, we analyzed normal, non-metastatic, and metastatic canine mammary tissues using histological approaches and label-free quantitative proteomics. Publicly available human breast cancer transcriptomic datasets were interrogated for *in silico* validation of conserved molecular signatures. Proteomic profiling identified 12 differentially expressed ECM-related proteins: eight were upregulated (COL12A1, COL4A1, COL4A2, SERPINH1, SERPINF1, HTRA1, TNC, PCOLCE) and four were downregulated (MMRN1, ABI3BP, DPT, OGN). The downregulated proteins were further validated in human breast cancer transcriptomes. Collectively, these findings indicate active ECM remodeling during tumor progression, characterized by increased expression of proteins associated with matrix stiffness and invasiveness. This study highlights evolutionarily conserved mechanisms of ECM dysregulation in breast cancer and identifies potential matrix targets for translational research and biomarker development.

## Introduction

Breast cancer is a leading cause of cancer-related mortality in women (1) and the most common neoplasm in female dogs (2, 3). Human and canine mammary tumors exhibit remarkable histopathological, genetic, molecular, and clinical similarities (4, 5). This resemblance supports the use of the canine model in translational research, given their spontaneous tumor development, intrinsic heterogeneity, and shared environmental and hormonal influences. Consequently, canine mammary tumors constitute a valuable alternative to experimentally induced murine models (6, 7).

The extracellular matrix (ECM) plays a central role in the dynamics of malignant progression (8, 9) once the ECM is continuously remodeled during tumorigenesis, directly influencing processes such as adhesion, proliferation, migration, angiogenesis, immune evasion, and therapy resistance (10, 11). Structural and compositional reorganization of the ECM sustains tumor biology, generates bioactive fragments and reprograms cellular signaling, thereby contributing to the establishment of a permissive metastatic niche (12, 13). Accordingly, ECM has emerged as a source of prognostic and therapeutic biomarkers. Matrix proteins such as tenascin-C, periostin, fibronectin, type I and IV collagens, and members of the SPARC family have been associated with poor clinical outcomes and therapeutic resistance in several cancers, including breast cancer (14, 15). The identification of such biomarkers supports risk stratification, definition of therapeutic targets and development of molecular imaging strategies (16).

Despite these advances, most studies have focused exclusively on human tumors, while spontaneous comparative models remain underexplored. A systematic analysis of the ECM in canine mammary cancer is still at an early stage, particularly with respect to the proteomic landscape across metastatic progression. Few studies have characterized ECM composition at distinct disease stages or evaluated its translational potential for biomarker discovery. Moreover, the lack of cross-validation with human data limits consolidation of the canine model as a robust comparative tool.

In this context, the present study investigated the extracellular matrix profile associated with metastatic progression in spontaneous mammary tumors of female dogs, with a particular focus on identifying differential ECM biomarkers. Through the integration of comparative proteomic analyses with *in silico* validation using human breast cancer transcriptomic datasets, we sought to identify conserved molecular targets and to advance the understanding of the ECM’s role in tumor biology, highlighting its potential as a source of clinically relevant biomarkers.

## Methods

### Animals, sample collection and ethical aspects

A total of 41 female dogs with histopathologically confirmed mammary carcinoma were included in this study. The cohort comprised animals of different breeds and ages, both intact and spayed, all of which underwent unilateral radical mastectomy between 2018 and 2019. Surgical procedures were performed by the Mammary Oncology Research Group at the Hospital of Veterinary Medicine Professor Renato Medeiros Neto (HOSPMEV/UFBA), Salvador, Bahia, Brazil.

Of the 41 samples included in the study, five corresponded to normal mammary tissue used as controls, while the remaining 36 samples were primary mammary carcinomas. Primary mammary tumors were collected for analysis, and the dogs were stratified into three experimental groups: 18 with non-metastatic mammary tumors, 18 with metastatic tumors, and 5 with normal mammary tissue. Normal samples were obtained from contralateral mammary glands of carcinoma-bearing dogs, specifically from regions without grossly visible tumor lesions.

All procedures were approved by the Animal Use Ethics Committee of the Gonçalo Moniz Institute, Fiocruz, under protocol number 07/2018.

### Anatomopathological study

The anatomopathological study was conducted at the Laboratory of Investigation in Global Health and Neglected Diseases, Gonçalo Moniz Institute (IGM/Fiocruz), in Salvador, Bahia, Brazil. For histopathological evaluation, mammary gland fragments were processed using standard paraffin-embedding protocols. Tissue sections of 4 µm thickness were prepared and stained with hematoxylin-eosin (HE).

Tumor classification was performed by a veterinary pathologist specializing in mammary oncology, following the guidelines of the Consensus for Diagnosis, Prognosis, and Treatment of Canine Mammary Tumors (17). *In situ* areas were defined by the presence of epithelial cells organized into tubular structures, associated with continuous layers of myoepithelial cells and an intact basement membrane, as demonstrated by HE staining (17).

### Special staining and histomorphometry analysis

Histological samples were subjected to special staining for the morphological characterization of the extracellular matrix (ECM) within the analyzed tissues.

Due to technical issues related to tissue processing, staining quality, or insufficient material for reliable quantification, some samples were excluded from histomorphometric analyses. As a result, the final dataset comprised 4 normal samples, 16 non-metastatic tumors, and 16 metastatic tumors.

Masson’s Trichrome staining was used to quantify the total collagen content in invasive areas and *in situ* lesions (18). Total marked area and the staining intensity were evaluated, with intensity quantified on a scale from 0 to 255 (with 0 and 255 corresponding to minimal and maximal staining, respectively).

Picrosirius Red staining allowed for the differentiation of collagen into types I and III under polarized light (19–21). For this analysis, areas of type I collagen, type III collagen, and the total area corresponding to the sum of both types (collagen I + III) were quantified separately.

The quantification of pixels for Masson’s Trichrome and Picrosirius Red stains was performed using a standardized semi-automatic method developed in our laboratory, utilizing ImageJ software, Fiji version 2.9.0, with the Versatile Wand Tool. Quantification was based on standardized semi-automatic histomorphometry, and group differences were inferred from the quantified areas rather than visual inspection alone.

### Extracellular matrix protein enrichment

For this stage, 15 samples were selected: five from non-metastatic mammary carcinomas, five from metastatic mammary carcinomas, and five from contralateral normal mammary tissue. Extracellular matrix (ECM) protein enrichment was performed through sequential extraction of tumor tissues cryopreserved at –80 °C, without the use of chemical fixatives.

This sequential extraction strategy was designed to progressively remove soluble, intracellular, and loosely associated proteins, thereby increasing the relative abundance of ECM components prior to proteomic analysis.

Frozen samples were sectioned into 20–50 mg fragments and washed five times with 1× PBS. Sequential extractions were then performed using different buffers, as previously described (22). Initially, NaCl buffer was added at a 10:1 (v/w) ratio, followed by mechanical agitation for 1 hour at 600 rpm at room temperature. This step primarily removes highly soluble and weakly associated proteins. After phase separation, the supernatant was collected and stored at –20 °C.

The remaining pellet was washed again with NaCl buffer and subsequently extracted with SDS buffer at the same 10:1 (v/w) ratio, under continuous agitation for 16 hours at 600 rpm and 20 °C. This extraction targets membrane-associated and residual cellular proteins. After separation, this extract was also stored.

The residual tissue was then washed with distilled water and extracted with guanidine hydrochloride (GuHCl) buffer at a 5:1 (v/w) ratio for 72 hours at 3,200 rpm at room temperature. This final step solubilizes highly cross-linked and insoluble ECM proteins. Following centrifugation, the final supernatant was collected and stored at –20 °C for subsequent analyses.

For proteomic profiling, only GuHCl extract, expected to be the most enriched in insoluble ECM components, was selected for LC–MS/MS analysis. NaCl and SDS extracts were stored for potential downstream applications but were not analyzed by mass spectrometry in the present study.

### Protein quantification

The protein lysate was measured using the tryptophan method (23). Briefly, samples were dissolved in 8 M urea containing 0.1 M Tris-HCl (pH 7.8), and tryptophan content was quantified by spectrofluorometry under the following conditions: excitation at 295 nm, emission at 350 nm, gain of 100, and a 7 mm optical path height. Five replicate readings were acquired for each sample.

### Sample preparation for proteomic analysis

Prior to mass spectrometry analysis, ECM-enriched protein extracts were subjected to SDS-PAGE as a sample preparation step to allow efficient in-gel digestion and to minimize interference from extraction reagents commonly used in extracellular matrix enrichment protocols.

The samples were subjected to SDS-PAGE, followed by in-gel digestion. Gels were sliced in small pieces and destaining in 50% ethanol, 50% ammonium bicarbonate (ABC) Disulfide bonds were reduced with 10 mM dithiothreitol (DTT) and incubation at 56 °C for 1 hour.

Next, gel was treated with 55 mM iodoacetamide (IAA) incubated for 1 hour at room temperature in the dark for alkylation of cysteine residues. Then, gel pieces were washed with ethanol followed by ABC, and this step was repeated. After this step, 12.5 ng/µl trypsin in ABC was added to cover the gel inside the tube, with overnight incubation at 37 °C.

In the following day, peptides were extracted from the gel pieces by twice incubations in 3% TFA, 30% acetonitrile and twice incubations in 100% acetonitrile. The sample volume was reduced in vacuum centrifuge, and the generated peptides were purified by reverse-phase chromatography using homemade C18 stage-tip. The stage-tips were pre-conditioned with 100% methanol and 0.1% formic acid. Samples were applied, followed by washing with 0.1% formic acid, and adsorbed peptides were eluted with 40% acetonitrile solution in 0.1% formic acid. The eluate was dried in vacuum centrifuge, resuspended in 0.1% formic acid and injected onto LC-MS/MS system.

### Mass spectrometry analysis

The samples were analyzed at Mass Spectrometry Facility (RPT02H), Technological Platforms Network FIOCRUZ, Oswaldo Cruz Foundation (FIOCRUZ) using an Orbitrap Fusion Lumos mass spectrometer (Thermo Scientific) coupled to an Ultimate 3000 RSLCnano (Thermo Scientific). Peptide separation was achieved using a 90-minute gradient at a flow rate of 250 nL/min. The elution gradient consisted of 5% to 40% solvent B (acetonitrile with 0.1% formic acid) over 90 minutes. The chromatographic system employed a 15 cm analytical column with 3 µm C18 particles (ReproSil-Pur 120 C18-AQ, Dr. Maisch) packed into a 75 µm (ID) × 360 µm (OD) FSC emitter. The mass spectrometer operated in Data Dependent Acquisition (DDA) mode with 2 s maximum per duty cycle, selecting the most intense precursor ions for Higher-Energy Collisional Dissociation (HCD) fragmentation and analysis in the orbitrap analyzer (MS/MS). Acquisition parameters included: nano-electrospray voltage set to 2.3 kV, injection times of 50 ms and resolution of 120,000 for MS1 (300–1500 m/z) and 22 ms and 15,000 for MS2. The dynamic exclusion time was set to 60 seconds. The internal calibration option was enabled for MS1 scans.

The mass spectrometry proteomics data have been deposited to the ProteomeXchange Consortium via the PRIDE (24, 25) partner repository with the dataset identifier PXD074203.

### Differentially expressed protein analysis

Proteomic statistical analyses were conducted based on label-free quantification (LFQ) of protein abundance, processed on the MaxQuant platform (version 2.2.0.0) using the Canis lupus familiaris reference proteome from UniProt (UP000805418, release 2023-09-20) with the MaxQuant contaminant database included. Identified proteins with a false discovery rate (FDR) ≤ 1% were filtered. The generated “proteinGroups.txt” table was imported into R (version 4.2) for the identification of differentially expressed proteins.

The DEP R package (differential enrichment analysis of proteomic data), version 1.26.0, was used to analyze differentially expressed proteins among the normal mammary gland, non-metastatic mammary tumor, and metastatic mammary tumor groups. Contaminant and reverse proteins were removed. The remaining data were filtered to include only proteins that showed an LFQ value > 0 in at least three samples. The resulting LFQ intensities were normalized and imputed using random draws from a Gaussian distribution centered at a minimum value (P < 0.05). Finally, differential enrichment analysis was performed in DEP using the Limma function, selecting proteins with adjusted P-values < 0.05 and log2 (expression change) > 1, respectively.

For confirmation of extracellular matrix proteins, the matrisome categorization, as proposed by Naba et al. (26) and available on the website (https://naba.lab.uic.edu/the-matrisome-project), was utilized.

### Functional enrichment analysis

Gene sets corresponding to the identified and differentially expressed matrix proteins were analyzed using g:Profiler tool (https://biit.cs.ut.ee/gprofiler), with default parameters and Canis familiaris as the reference species.

The results obtained were filtered based on adjusted statistical significance and term size, prioritizing those most representative and biologically relevant. Redundancy among terms was reduced using REVIGO tool (http://revigo.irb.hr/), with default settings. Subsequently, the selected terms were used for the construction of visualizations and functional interpretations.

### Acquisition and categorization of TCGA expression and clinical data

Gene expression data (RNA-Seq, HTSeq-counts format) and clinical information for breast cancer (BRCA) were obtained from The Cancer Genome Atlas (TCGA) via the Genomic Data Commons (GDC, https://portal.gdc.cancer.gov/), accessed in March 2025. Samples from two TCGA studies were included: “TCGA-BRCA” (Study ID: brca_tcga_gdc) and “Pan-Cancer Atlas Breast Cancer cohort” (Study ID: brca_tcga_pan_can_atlas_2018).

Samples were categorized into three experimental groups: normal breast tissue, tumor without metastasis, and tumor with metastasis. Group classification was based on clinical variables available in the TCGA metadata, specifically “AJCC Pathologic N-Stage”, “AJCC Pathologic M-Stage”, and “Neoplasm Disease Stage American Joint Committee on Cancer Code”. The metastasis group included samples from patients with evidence of regional lymph node involvement (N positive) and/or distant metastasis (M positive) at the time of diagnosis. Samples from patients without regional lymph node metastasis (N0) and without distant metastasis (M0) were assigned to the non-metastasis group. Additionally, samples from non-tumoral breast tissue were included as the normal control group.

Data download and preprocessing were performed using the TCGAbiolinks package within the R environment (version 2.37.1).

### Differential gene expression and pathway enrichment analysis

Differential gene expression analysis was conducted using the DESeq2 package (version 1.44.0) in R. Gene annotation was based on GENCODE (GRCh38.p14, release 46; https://www.gencodegenes.org/) retaining only protein-coding genes. Raw counts were imported into a DESeqDataSet (DESeq2; (27)) with a design including non-tumor, metastatic, and non-metastatic samples. Genes with normalized counts ≤10 in the minimum group size were excluded. Data were normalized with estimateSizeFactors and variance-stabilized (VST).

The statistical design considered the three experimental groups (normal tissue, tumor without metastasis, and tumor with metastasis). Differential expression was assessed through pairwise comparisons between the groups: (i) tumor without metastasis vs. normal tissue, (ii) tumor with metastasis vs. normal tissue, and (iii) tumor with metastasis vs. tumor without metastasis. The magnitude of differential expression was calculated as the log2-transformed fold change (log2FC), and statistical significance was determined using the Wald test implemented in DESeq2.

P-values were adjusted for multiple testing using the Benjamini-Hochberg false discovery rate (FDR) correction. Genes with FDR-adjusted P-values < 0.05 and |log2 fold change| ≥ 2 were considered differentially expressed genes (DEGs).

### Statistical analysis

Statistical analyses for identifying differentially expressed proteins and transcriptomic gene expression and pathway enrichment were performed as detailed in the sections “Differentially Expressed Protein Analysis” and “Differential Gene Expression and Pathway Enrichment Analysis,” respectively. Additional statistical analyses were conducted as described below.

To evaluate other quantitative variables among the experimental groups, data normality was assessed using the Shapiro-Wilk test. For non-parametric data, Kruskal-Wallis’s test was employed, followed by Dunn’s *post hoc* test for multiple comparisons. The relationship between continuous variables was evaluated using Spearman’s correlation coefficient.

All statistical analyses were carried out using R (version 4.2). A P-value of < 0.05 was considered statistically significant.

### Ethical statement

This study utilized publicly available data retrieved from the cBioPortal platform (https://www.cbioportal.org), specifically from The Cancer Genome Atlas (TCGA-BRCA) database. All datasets were coded and anonymized, precluding any possibility of participant identification. Accordingly, the use, analysis, and reporting of these data involve no ethical concerns. The authors declare no economic or institutional conflicts of interest related to the methods applied or the results presented herein.

## Results

### Mammary specimens

Mammary specimens were collected from 41 female dogs, including animals diagnosed with mammary neoplasms and contralateral normal mammary tissue used as controls, ranging in age from 6 to 16 years, with an overall average of 11 years. The most frequent breeds were Mixed-Breed (MD) and Poodle, representing 39% (n=16) and 29% (n=12), respectively. The remaining 32% (n=13) comprised Yorkshire (n=2), Rottweiler (n=2), Pinscher (n=1), Dachshund (n=1), Pitbull (n=1), French Bulldog (n=1), Akita Inu (n=1), Cocker Spaniel (n=1), Labrador (n=1), and Maltese (n=1).

### Clinicopathological evaluation (staging and grade)

Histopathological analysis was performed on 36 of the 41 collected mammary specimens. Among the 36 analyzed cases of mammary carcinoma, the majority were classified as carcinoma in mixed tumor (55.6%; n=20) or carcinosarcoma (22.2%; n=8), while the remaining cases (22.2%; n=8) exhibited variant histological subtypes. Regarding metastasis, 50% (n=18) had distant or lymphatic metastasis of mammary carcinoma, and 50% (n=18) showed no clinically detectable metastasis at the time of surgery.

In terms of clinical staging, most dogs with metastasis (17/18; 95%) were classified as stage IV, with histological grade II (12/18; 67%). In contrast, most dogs without metastasis presented as stage III (9/18; 50%) with histological grade I (10/18; 56%). Complete clinicopathological data are summarized in **Table 1**.

**Table 1:** Clinicopathological characteristics of canine mammary neoplasm cases, according to metastatic status.

Characteristics	Metastatic group (n=18/36; 50%)	Non-Metastatic group (n=18/36; 50%)
Mean Age (years)	11	11
Tumor Size (cm)		
< 3 cm	0/18 (0%)	0/18 (0%)
3 cm < x < 5 cm	4/18 (22%)	7/18 (39%)
> 5 cm	14/18 (78%)	11/18 (61%)
Clinical Staging		
I	0/18 (0%)	1/18 (6%)
II	0/18 (0%)	8/18 (44%)
III	0/18 (0%)	9/18 (50%)
IV	17/18 (95%)	0/18 (0%)
V	1/18 (5%)	0/18 (0%)
Histological Grade		
I	2/18 (11%)	10/18 (55%)
II	12/18 (67%)	5/18 (28%)
III	4/18 (22%)	3/18 (17%)
Histological Subtype		
Solid	2/18 (11%)	0/18 (0%)
Tubular	1/18 (6%)	1/18 (6%)
Ductal	1/18 (6%)	0/18 (0%)
CSS*	2/18 (11%)	6/18 (33%)
CTM*	9/18 (50%)	11/18 (61%)
STM*	1/18 (6%)	0/18 (0%)
Papillary	2/18 (10%)	0/18 (0%)

CSS, Carcinosarcoma; CTM, Carcinoma in mixed tumor; STM, Sarcoma in mixed tumor.

### Morphometric analysis with special staining

**Figure 1:**
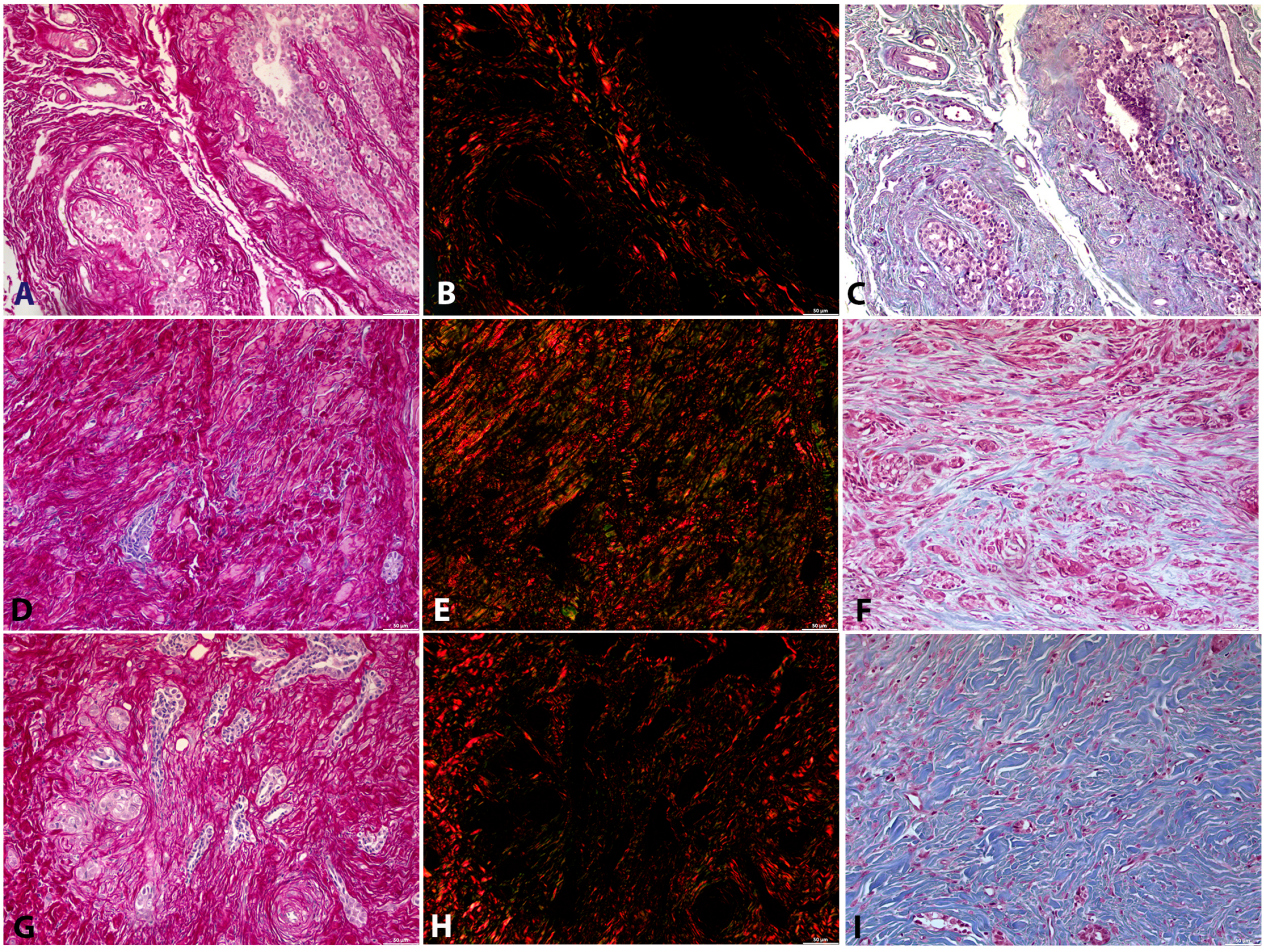
Morphological characterization of the extracellular matrix (ECM) using Masson’s trichrome and picrosirius red special stains. Masson’s trichrome highlights collagen fibers in blue, whereas picrosirius red staining, under polarized light, distinguishes type I collagen fibers (red) from type III collagen fibers (green). **(A–C)** Normal mammary gland: **(A)** picrosirius red, bright field; **(B)** picrosirius red, polarized light; **(C)** Masson’s trichrome. **(D–F)** Non-metastatic mammary carcinoma: **(D)** picrosirius red, bright field; **(E)** picrosirius red, polarized light; **(F)** Masson’s trichrome. **(G–I)** Metastatic mammary carcinoma: **(G)** picrosirius red, bright field; **(H)** picrosirius red, polarized light; **(I)** Masson’s trichrome.

For the morphological characterization of the extracellular matrix, Masson’s trichrome and picrosirius red stains were employed. Representative images from ten fields per case were acquired following whole-slide scanning (**Figure 1**) and analyzed using ImageJ software. The variables evaluated were collagen area and staining intensity. Picrosirius red staining enabled the differentiation between type I and type III collagen, whereas Masson’s trichrome staining allowed quantification of the total ECM area and its staining intensity. These analyses provided quantitative data on collagen deposition in metastatic and non-metastatic groups.

**Figure 2:**
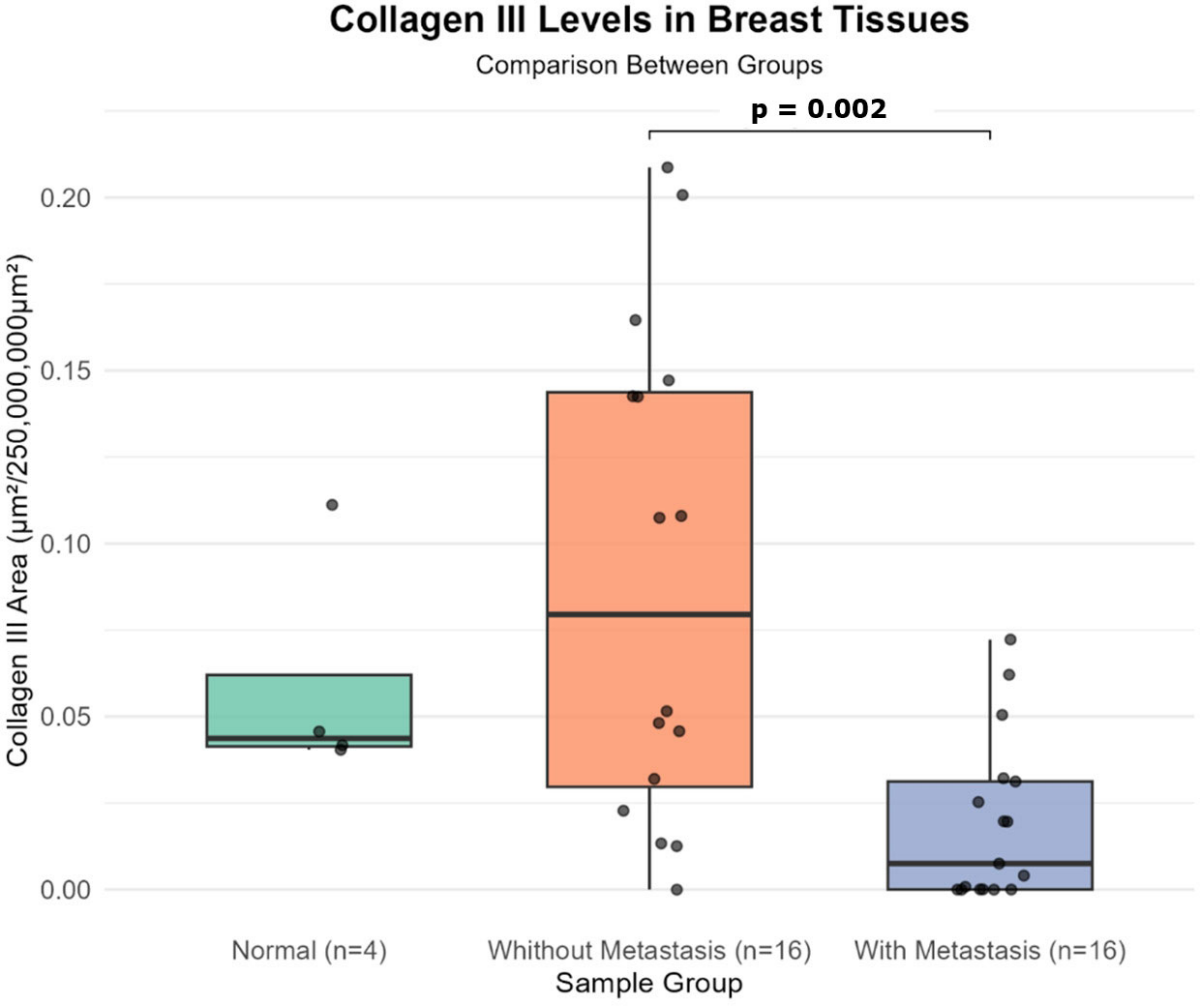
Distribution of type III collagen in mammary tissue samples. Boxplots represent the median, interquartile range, and outliers for the normal (n = 4), non-metastatic (n = 16), and metastatic (n = 16) groups. A significant reduction in type III collagen was observed in the metastatic group compared with the non-metastatic group (p = 0.002; Dunn’s *post hoc* test with Benjamini–Hochberg correction).

Statistical analysis revealed significant differences in the distribution of type III collagen area among the metastatic group, the non-metastatic group, and a control group consisting of normal mammary tissue (Kruskal-Wallis: χ2 = 11.996; df=2; p=0.002). To further explore these differences, Dunn’s *post hoc* test with Benjamini–Hochberg correction for multiple comparisons was applied. The metastatic group demonstrated a significantly lower type III collagen content compared with the non-metastatic group (z = –3.349; p = 0.002). These findings are illustrated in **Figure 2**.

### Protein analysis

**Figure 3:**
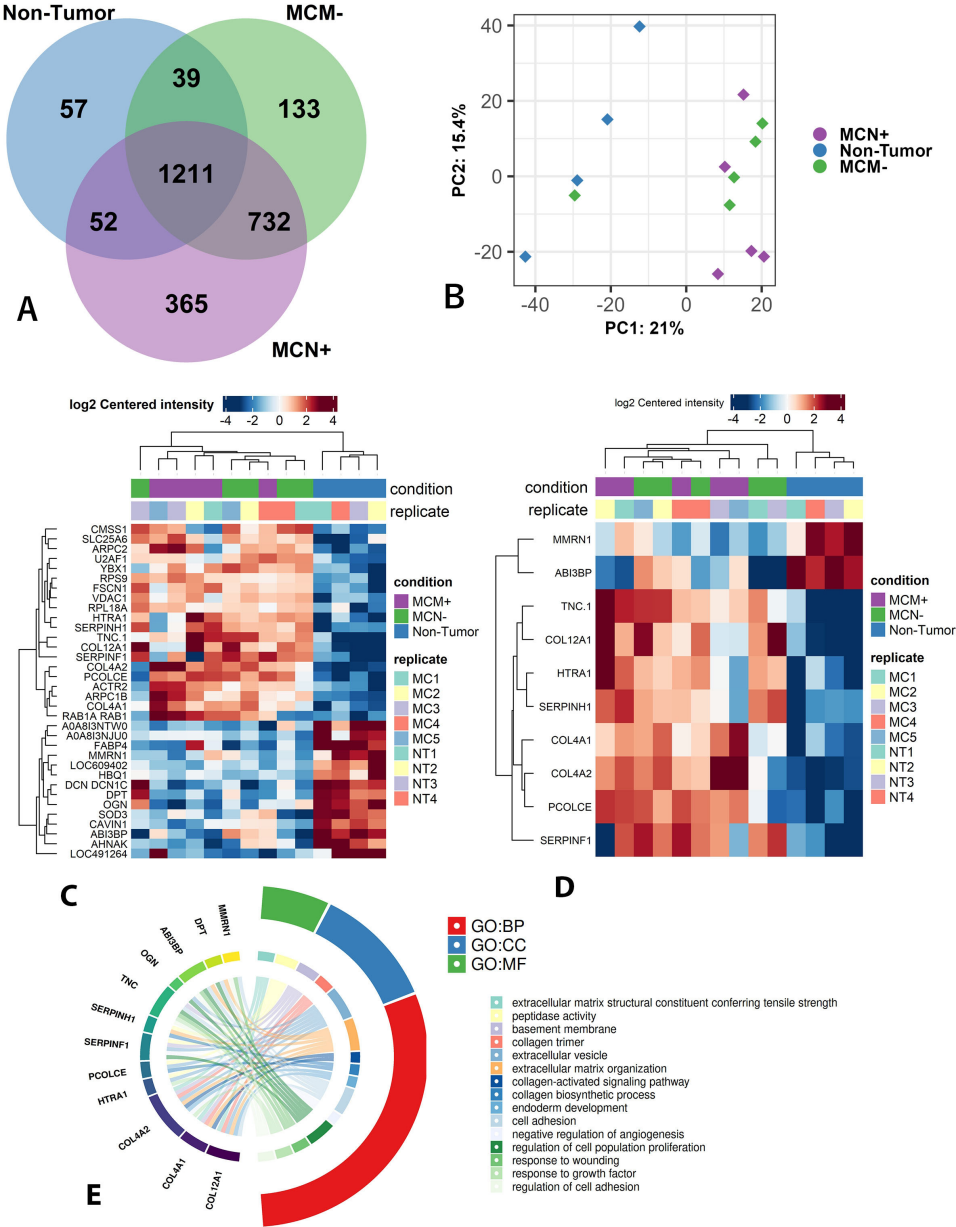
Overview of proteomic analysis. **(A)** Venn diagram representing the number of proteins identified in the raw proteomic dataset that are unique to each group or shared among groups. **(B)** Principal component analysis (PCA) showing separation between groups based on global protein profiles: normal tissue (Non-Tumor), non-metastatic carcinoma (MCM−), and metastatic carcinoma (MCN+). **(C)** Heatmap of the 34 proteins differentially expressed between the groups: metastatic tumor (MCN+), non-metastatic tumor (MCM−), and normal tissue (Non-Tumor). **(D)** Heatmap of 12 extracellular matrix (matrisome) proteins identified among the 34 differentially expressed proteins. **(E)** Functional enrichment analysis of differentially expressed extracellular matrix proteins, categorized by BP, biological process; CC, cellular component; MF, molecular function.

Proteomic analysis of canine mammary tissues was performed on 15 samples (5 control, 5 non-metastatic, and 5 metastatic), identifying a total of 2,589 proteins in the raw dataset (**Figure 3A**). One normal sample was excluded due to the absence of protein quantification, resulting in 14 samples retained for downstream analyses. After quality filtering and noise reduction, 1,358 proteins were deemed reliably quantified and used for subsequent comparative analyses (**Supplementary File 1**). Principal Component Analysis (PCA) (**Figure 3B**) revealed a clear separation between the normal and tumor groups along principal component 1 (PC1), which accounted for 20.9% of the variance. In contrast, non-metastatic and metastatic groups exhibited partial overlap, although a trend toward separation was observed.

In the differential expression analysis (considering an adjusted p-value threshold < 0.05 and fold change >1.0), 34 proteins were identified as differentially expressed among the groups. The performed comparisons were normal group vs. non-metastatic group, normal group vs. metastatic group, and non-metastatic group vs. metastatic group. The first two comparisons resulted in 27 and 18 differentially expressed proteins, respectively. Interestingly, the comparison between the two tumor groups (non-metastatic vs. metastatic) did not reveal any statistically differentially expressed proteins. A heatmap demonstrated distinct protein expression profiles in the normal group compared with both tumor groups (**Figure 3C**).

**Figure 4:**
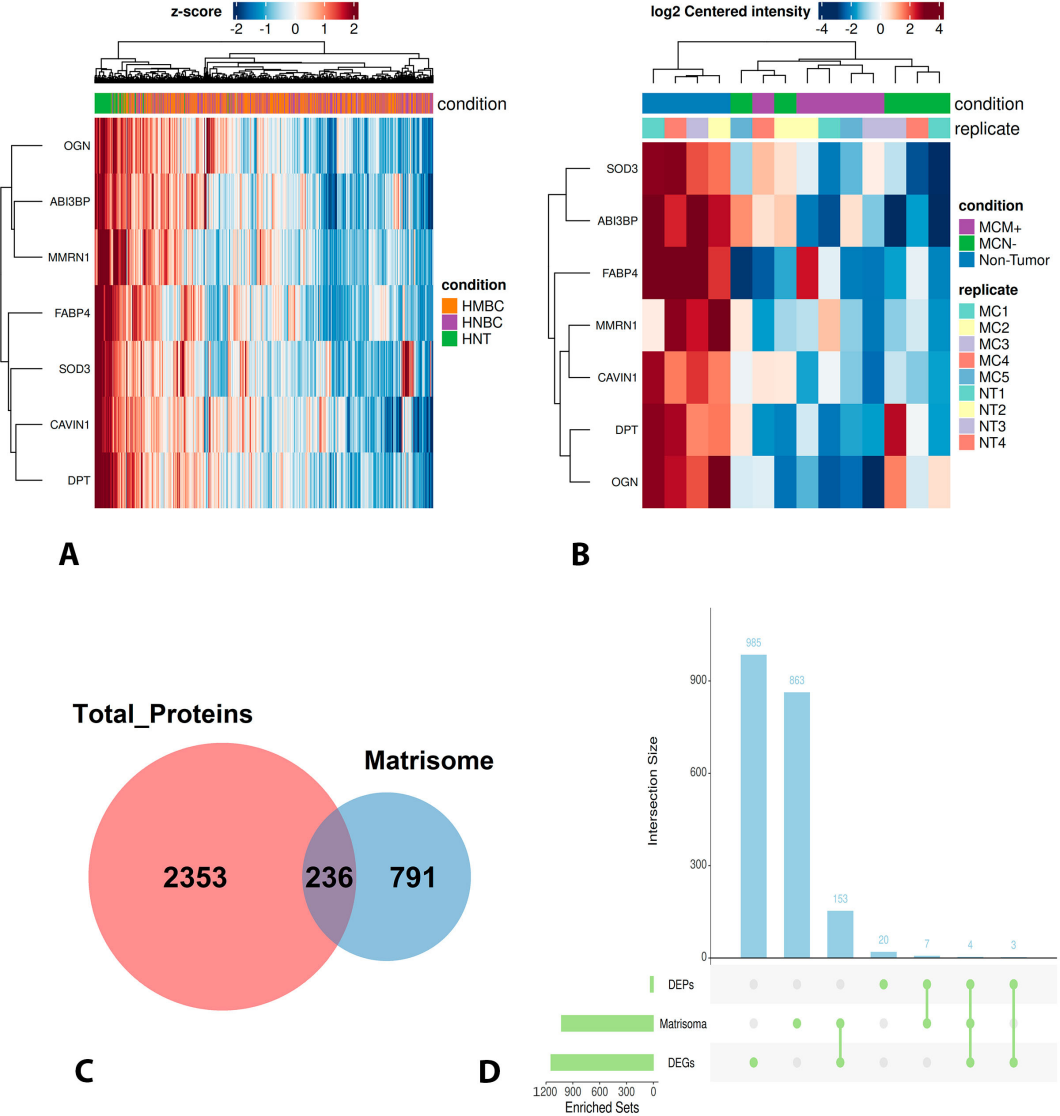
Integration of transcriptomic, proteomic, and matrisome analyses. **(A)** Heatmap of gene expression derived from human breast cancer transcriptomic data (TCGA) for genes corresponding to proteins identified as differentially expressed in the canine proteomic analysis. **(B)** Heatmap of protein expression for the same targets identified in the canine proteomic dataset. **(C)** Venn diagram illustrating the overlap between all proteins identified in the raw proteomic analysis and extracellular matrix (matrisome) proteins curated from the Matrisome Project database. **(D)** Proteins located at the intersection of proteomic, transcriptomic, and matrisome analyses, representing conserved extracellular matrix–related targets across species.

The protein extraction method was specifically optimized for extracellular matrix enrichment. In the raw proteomic dataset, 236 proteins overlapped with the Matrisome database (**Figure 4C**). After quality filtering, 1,358 proteins remained reliably quantified, among which 175 were classified as ECM components, corresponding to approximately 13% of the quantified proteome. Among the 34 differentially expressed proteins, 12 were ECM-related: MMRN1, ABI3BP, DPT and OGN were downregulated in tumor groups compared with the normal group, whereas TNC, COL12A1, HTRA1, SERPINH1, COL4A1, COL4A2, PCOLCE, and SERPINF1 were upregulated (compared to the normal group). A heatmap of these ECM-proteins further highlighted the clear segregation of the normal group from the tumor groups based on expression profiles (**Figure 3D**). Functional enrichment specific to these 12 ECM proteins is shown in **Figure 3E**. These ECM candidates were subsequently assessed for cross-species conservation through in silico validation in publicly available human breast cancer transcriptomic datasets (TCGA).

### Transcriptomic analysis

Using publicly available human breast cancer data from TCGA, three experimental groups were defined: 214 normal mammary gland samples, 908 non-metastatic tumor samples, and 918 metastatic tumor samples. These transcriptomic data were used exclusively for *in silico* validation of candidates identified in the canine proteomic analysis.

In the canine proteomic dataset, 34 proteins were identified as differentially expressed among normal tissue, non-metastatic tumors, and metastatic tumors, of which 12 were classified as extracellular matrix components. Cross-species comparison revealed that 7 of these 34 proteins showed concordant differential gene expression in the TCGA transcriptomic dataset. Notably, four ECM-related genes: MMRN1, DPT, OGN, and ABI3BP were consistently downregulated at both the protein level in canine tumors and the transcript level in human breast cancer samples. Heatmaps illustrate the expression patterns of the shared targets at the protein (canine) and gene (human) levels are presented in **Figures 4A, B**. The overlap between proteomic, transcriptomic, and matrisome datasets is summarized in **Figure 4D**.

## Discussion

In this study, the extracellular matrix (ECM) protein profile was evaluated across three groups relevant to canine tumor progression: normal tissue, non-metastatic mammary carcinoma, and metastatic mammary carcinoma. Proteomic analyses revealed a clear separation between normal and tumor samples, whereas no evident distinction was observed between metastatic and non-metastatic tumors. This pattern was consistently detected in the principal component analysis (PCA) and heatmaps derived from the canine proteomic dataset.

A similar trend was observed during in silico validation using human breast cancer transcriptomic data, in which gene expression heatmaps of targets corresponding to the differentially expressed proteins likewise showed limited segregation between metastatic and non-metastatic tumors. Such findings are not unexpected, given the pronounced heterogeneity of breast cancer, which encompasses multiple histological and molecular subtypes (28, 29). Owing to the exploratory design of this study, mammary carcinoma samples were included in both proteomic and transcriptomic analyses irrespective of subtype, resulting in a highly heterogeneous dataset and, consequently, diverse gene and protein expression profiles.

In the morphological analysis, a positive correlation was identified between type III collagen levels and the groups evaluated. The reduction of type III collagen in metastatic tumors compared with non-metastatic tumors reinforces previous evidence that a shift from compliant to stiffer collagen networks creates a more permissive microenvironment to cellular invasion and disease progression (30, 31). Consistent with our findings, several studies have reported decreased type III collagen levels in more aggressive breast cancer contexts (32, 33). However, the unequal number of samples across groups, 4 in the normal group, 16 in the non-metastatic group, and 16 in the metastatic group, represents a limitation that may have reduced the statistical power to detect significant differences between normal and tumor tissues.

A total of twelve ECM proteins were differentially expressed between tumors and normal tissue: eight proteins were upregulated and four were downregulated. Notably, these four proteins (MMRN1, ABI3BP, DPT, and OGN) displayed a similar expression pattern in the human transcriptomic analysis, highlighting concordance between the two omics approaches and coordinated regulation at both the RNA and protein levels.

MMRN1 is a protein involved in hemostasis and coagulation processes and, when incorporated into the ECM, may contribute to cell adhesion (34). It has been proposed as a potential biomarker in several cancer types due to its differential expression in malignant versus normal tissues. In breast cancer, reduced MMRN1 expression has been associated with greater disease severity and decreased patient survival (35–38).

ABI3BP is an ECM protein that regulates cell proliferation, differentiation, and signal transduction in various tissues (39, 40). It functions as a tumor suppressor in several cancers by negatively modulating oncogenic signaling pathways (38, 40). Reported activities include induction of cell cycle arrest, inhibition of epithelial–mesenchymal transition (EMT), and reduction of invasive potential. In breast cancer, both ABI3BP mRNA and protein levels are reduced compared with normal tissue. Furthermore, ABI3BP has been implicated as an important gene in the antitumor immune response (41, 42).

DPT is an ECM protein that typically accelerates collagen fibrillogenesis and interacts with TGF-β1. In breast cancer, DPT expression is significantly reduced compared to normal tissues and correlates with clinical features such as age, histological type, and stage. Overexpression of DPT inhibits the proliferation, migration, and invasion of breast cancer cells, acting as a tumor suppressor by repressing the PI3K/Akt signaling pathway (43, 44).

OGN is a small leucine-rich proteoglycan (SLRP) of the extracellular matrix, involved in ECM organization and cellular signal transduction. In breast cancer, OGN is generally downregulated in tumor tissue, and its low expression is associated with poorer prognosis and more aggressive subtypes (45, 46). OGN functions as a tumor suppressor by inhibiting proliferation and invasion through suppression of the PI3K/Akt/mTOR pathway and reversal of EMT (45). Interestingly, OGN is also highly expressed in dormant cells, and its expression in primary tumors has been shown to predict longer recurrence-free survival (47).

The overexpression of COL12A1, SERPINH1, PCOLCE, COL4A1, and COL4A2 indicates an active process of ECM remodeling, characterized by increased density, stiffness, and fibrillar complexity. Type XII collagen (COL12A1), a member of the fibril-associated collagens with interrupted triple helices (FACIT) family, interacts with type I collagen to regulate the spatial orientation and three-dimensional organization of the ECM. Progressive upregulation of COL12A1 during tumor progression, previously reported in multiple models including human breast cancer, has been associated with cancer-associated fibroblasts (CAFs), TGF-β1 activation, enhanced tissue stiffness, and M2 macrophage infiltration—factors strongly linked to increased tumor aggressiveness and therapeutic resistance (48–50).

Elevated levels of SERPINH1 (Hsp47), a molecular chaperone essential for collagen folding and secretion, further support the presence of a highly productive and organized ECM (51, 52). SERPINH1 is directly associated with excessive deposition of type I and IV collagens, increased tissue stiffness, and activation of pro-metastatic signaling pathways (53). Functional studies have demonstrated that SERPINH1 inhibition reduces invasiveness, EMT, and colony-forming ability in tumor models, underscoring its dual role as both a structural and functional regulator of the ECM (54–56).

PCOLCE, a protein that enhances the activity of the BMP1 protease during procollagen maturation, was also found to be overexpressed. Its function ensures efficient production of mature collagen, which is essential for maintaining the architecture of the tumor matrix (57). Omics studies have reported higher PCOLCE expression in several cancer types, including breast cancer, compared with normal tissues. PCOLCE appears to influence tumor growth and metastatic progression through the PI3K–Akt pathway and has been correlated with cancer-associated fibroblast (CAF) markers and unfavorable clinical outcomes (58).

In the context of the basement membrane, the upregulation of COL4A1 and COL4A2 indicates active ECM remodeling. Although type IV collagens are traditionally regarded as structural barriers to invasion, they may undergo disordered reorganization in tumor tissues, generating bioactive fragments such as canstatin and tumstatin. These fragments can exert paradoxical effects on angiogenesis, apoptosis, and invasiveness, with the potential to either promote or inhibit tumor progression (59–61). Moreover, aberrant deposition of type IV collagens may reflect stromal attempts to restore tissue integrity in response to tumor aggression or provide a substrate for directed cell migration (62, 63).

Regarding matricellular and regulatory proteins, Tenascin-C (TNC) stands out as a multifunctional key element. TNC mediates cell–matrix communication, promotes EMT, activates tumor stem cell signaling pathways such as WNT and NOTCH, and contributes to immune evasion and metastatic niche formation (64). Its elevated expression is strongly associated with the presence of CAFs and with mechanical remodeling of the ECM, thereby promoting tumor cell survival and dissemination. In human breast cancer, high TNC levels have been linked to increased metastatic potential, particularly to the lungs (65). In addition, TNC actively participates in ECM remodeling through the regulation of matrix metalloproteinases, further contributing to tumor progression (66, 67).

Despite the overexpression of HTRA1 observed in this study, its behavior in aggressive tumor contexts is often the opposite, with reduced expression reported in malignant breast tissues. Commonly described as a protein with tumor-suppressive functions, HTRA1 downregulation has been associated with chemoresistance and decreased survival in breast cancer patients (68, 69). Nevertheless, HTRA1 also functions as a protease involved in extracellular matrix (ECM) cleavage, regulation of cell adhesion, and protein quality control. Its increased expression in this context may reflect an alternative functional role, potentially related to dynamic ECM remodeling, or may be attributable to specific cellular subpopulations within the tumor microenvironment (70, 71).

Similarly, SERPINF1 (PEDF) showed higher expression in tumor tissues compared with normal tissue, a finding that contrasts with prevailing literature describing PEDF as a tumor suppressor typically downregulated in malignancies (72, 73). Although loss of PEDF expression in cancer cells has been linked to poor prognosis and metastasis (74), it is important to note that PEDF can also be produced by stromal components of the tumor microenvironment, including fibroblasts (73). Furthermore, pan-cancer analyses suggest that elevated SERPINF1 expression may be associated with immune infiltration in the tumor microenvironment, indicating that its role may be highly context-dependent (75).

A discrepancy was observed between morphological and omics-based analyses. Quantification of special stains indicated a reduction of type III collagen in tumors, whereas proteomic and transcriptomic data revealed no significant alterations in the corresponding protein or gene expression. This inconsistency may reflect methodological differences: collagen deposition in the ECM represents the cumulative outcome of synthesis, degradation, and post-translational modifications over time, while molecular analyses capture only the transient state of gene or protein expression (8).

This divergence underscores a principal limitation of the study: the intrinsic heterogeneity of canine mammary carcinoma. The inclusion of multiple histological and molecular subtypes within the exploratory cohort likely limited the ability to distinguish ECM proteomic profiles between non-metastatic and metastatic tumors, consistent with the partial overlap observed in principal component and heatmap analyses. Furthermore, the lack of concordance between histomorphometric and molecular findings for type III collagen highlights the complexity of the ECM, where tissue deposition does not necessarily parallel contemporaneous gene or protein expression.

Importantly, some limitations of this study should be acknowledged. The relatively limited number of samples may restrict statistical power and limit the generalizability of the findings. Moreover, proteomic analyses were performed on bulk tissue samples, which precludes the resolution of cell type–specific contributions to extracellular matrix remodeling. Cross-species validation relied primarily on transcriptomic data from human breast cancer cohorts, which does not necessarily reflect protein-level regulation and should therefore be interpreted as in silico validation rather than direct functional confirmation. In addition, functional assays were not performed to directly interrogate the mechanistic roles of the identified ECM proteins in tumor progression and metastasis. Finally, although canine mammary carcinoma represents a valuable comparative model for human breast cancer, species-specific biological differences may influence the translational applicability of certain ECM-related targets.

Despite these constraints, the study achieved its primary objectives and provides a meaningful contribution to the understanding of ECM dynamics in breast cancer progression, offering a solid foundation for validation in larger, subtype-stratified cohorts and for future studies integrating spatially resolved and functional approaches to dissect ECM-driven mechanisms.

## Conclusion

In conclusion, the proteomic findings reveal a complex and dynamic remodeling of the extracellular matrix (ECM) in breast cancer, characterized by the predominant overexpression of structural and regulatory proteins associated with tumor progression. The coordinated upregulation of COL12A1, SERPINH1, PCOLCE, COL4A1, COL4A2, and TNC reflects a restructured tumor microenvironment that fosters invasion, metastasis, and therapeutic resistance. Conversely, the downregulation of MMRN1, ABI3BP, OGN, and DPT, proteins with well-recognized tumor-suppressive functions, underscores the disruption of stromal homeostasis. Collectively, these results emphasize the central role of the ECM in breast cancer pathophysiology and highlight promising molecular targets for therapeutic strategies aimed at modulating stromal architecture and mechanical properties. Furthermore, the canine model provides valuable insights into conserved mechanisms of ECM remodeling in mammary tumors, reinforcing its relevance as a translational platform for human breast cancer research.

## Data Availability

The datasets presented in this study can be found in online repositories. The names of the repository/repositories and accession number(s) can be found in the article/**Supplementary Material**.
